# Critical combination of initial markers for predicting refractory *Mycoplasma pneumoniae* pneumonia in children: a case control study

**DOI:** 10.1186/s12931-019-1152-5

**Published:** 2019-08-23

**Authors:** Young-Jin Choi, Ju-Hee Jeon, Jae-Won Oh

**Affiliations:** 1 Department of Pediatrics, College of Medicine, Hanyang University Guri Hospital, 249-1 Kyomun-Dong, Guri, Kyunggi-Do, Seoul, 471-701 South Korea; 2 Department of Medicine, Graduate School of Hanyang University, Seoul, South Korea

**Keywords:** Biomarkers, *Mycoplasma pneumoniae*, Refractory *Mycoplasma pneumoniae* pneumonia

## Abstract

**Background:**

It is unclear whether the responses of refractory and common *Mycoplasma pneumoniae* (MP) pneumonia to macrolides differ. Hence, this study aimed to identify biomarkers that may be used to distinguish refractory and common pneumonias caused by MP in children at hospital admission.

**Methods:**

The study included 123 children divided into five groups according to infection agent and treatment protocol: Group I included those with MP infection without documented viral infection, treated with only macrolides; Group II included those with MP infection without documented viral infection, treated with a combination of macrolides and methylprednisolone; Group III included those with MP infection and documented viral infection, treated with only macrolides; Group IV included those with viral pneumonia without documented MP infection; Group V was the control group composed of admitted children without MP or a documented viral infection. These five groups were further subdivided into Groups A (including Groups I, III, IV, and V) and B (Group II) according to the responses to macrolide treatment. Concentrations of cytokines interleukin 6, interleukin 17, interleukin 18, and tumor necrosis factor-α, and lactate dehydrogenase, and ferritin of all children were evaluated, and these levels were compared among the groups. Statistical comparisons were made using Kruskal Wallis test and Mann-Whitney U test.

**Results:**

Serum lactate dehydrogenase, interleukin 18, and ferritin concentrations were significantly higher in Group II than in Groups I, III, IV, and V and were significantly higher in Group B than in Group A. When the serum lactate dehydrogenase concentration was 350 IU/L or higher, the sensitivity and specificity for diagnosing refractory MP pneumonia were 73 and 80%, respectively. When the interleukin 18 level was 360 pg/mL or higher, the sensitivity and specificity for diagnosing refractory MP pneumonia were 93 and 70%, respectively. When the ferritin level was 230 pg/mL or higher, the sensitivity and specificity for diagnosing refractory MP pneumonia were 67 and 67%, respectively.

**Conclusion:**

These results suggest that serum lactate dehydrogenase, interleukin 18, and ferritin constitute the critical combination of biomarkers useful for predicting refractory MP pneumonia in children at hospital admission.

## Background

Pneumonia caused by *Mycoplasma pneumoniae* (MP) is usually a benign, self-limited disease that can be treated effectively with macrolides. However, in some pediatric cases, it may develop into a severe, life-threatening infection, resistant to conventional macrolide treatment.[1, 2] Refractory MP pneumonia is characterized by prolonged fever accompanied by deteriorating radiological findings; it does not respond to appropriate macrolide therapy. Glucocorticoid may be effective and well tolerated these cases. [3–5]

In MP pneumonia, the host’s cell-mediated immunity plays a key role in the development of pulmonary lesions. Studies of immunocompromised hosts suggest that T cells play a role in the pathogenesis of mycoplasma infection. This is further supported by an apparent correlation between the development of a delayed hypersensitivity skin reaction to MP infection and the severity of disease. [6, 7] In some cases of MP pneumonia, an active host immune response promoting the release of cytokines and a T-helper (Th)-1-mediated immune response may contribute to severe pulmonary injury. Furthermore, MP pneumonia may be related to mononuclear cell infiltration into the airway, which is mainly composed of CD4+ T cells, contributing to substantial amplification of the immune response and subsequent injury to the lung parenchyma. [8–10]

Lactate dehydrogenase (LDH) is an oxidoreductase that catalyzes the interconversion of pyruvate and lactate with concomitant interconversion of NADH and NAD+. It is occasionally termed as hydroxybutyrate dehydrogenase, as it also catalyzes the oxidation of hydroxybutyrate. This cytoplasmic enzyme is present in all major organs, including the brain, kidneys, liver, myocardium, and lungs. When cell lysis occurs or cell membranes are damaged, LDH is released into the extracellular space. Therefore, LDH levels can be used as a surrogate marker for tissue breakdown. Previous studies have shown that serum LDH levels are increased in patients with refractory MP pneumonia, including children requiring steroid therapy. [5, 11–16] We propose that LDH can be used as an initial indicator of refractory MP pneumonia, although further studies are necessary to confirm the usefulness of serum LDH level as an indicator of severity and the need for steroid therapy in MP pneumonia.

Th17 cells play a potent proinflammatory role in the immune system by producing the signature cytokine interleukin 17 (IL-17) and, to a lesser extent, other inflammation mediators including interleukin 6 (IL-6) and tumor necrosis factor-α (TNF-α). In addition to their effector function in the defense against extracellular pathogens, Th17 cells promote many inflammatory conditions, including several lung diseases such as chronic obstructive pulmonary disease. In mice inoculated with live MP, bronchoalveolar lavage fluid increases both IL-17 concentration and neutrophil counts. Similarly, IL-17-linked signal activation has been observed in patients with MP pneumonia who showed significantly higher levels of serum IL-17 than those with streptococcal pneumonia. Since IL-17 has been found to play a role in the transition from innate immunity to adaptive immunity, these observations lead to the hypothesis that some components of the MP extract induce IL-17, which in turn causes the excessive inflammatory cell reactions observed in MP pneumonia patients. [14–17]

Interleukin 18 (IL-18) is a proinflammatory cytokine that promotes Th1 cytokine responses. Recent studies have revealed elevated levels of serum IL-18 in children with MP pneumonia; the levels rose during the acute phase of the infection. [18, 19] Ferritin is induced by activated macrophages, which produce TNF-α. In the early stages of infection, phagocytic cells are stimulated to synthetize and release cytokines, which in turn stimulate the synthesis of ferritin. Increased serum levels of ferritin exert an immunosuppressive effect through the inhibition of lymphocyte proliferation. Excess ferritin also reflects increased levels of TNF-α, an important apoptotic factor, and of cytotoxic markers such as aspartate aminotransferase, LDH, and creatine kinase, which can indicate increased apoptosis. [14–19]

The mechanisms underlying the effect of steroids on MP pneumonia remain unknown. The rapid improvement of clinical symptoms and pulmonary lesions following steroid administration suggests that an increase in the related active cytokines and the resultant immunological dysregulation may play a crucial role. Recent evidence indicates that a pathogenesis similar to that of MP is responsible for the development of acute respiratory distress syndrome and multi-organ failure in severe viral infections such as severe acute respiratory syndrome. [15, 16] These viral diseases share some common laboratory findings, such as high serum levels of ferritin and LDH, similar to those we observed in patients with refractory MP pneumonia. [15–18, 20]

Accumulated evidence has shown that high-dose corticosteroids are effective in patients with MP infections. Combination therapy with macrolides and systemic steroids is more effective in reducing MP-induced pulmonary inflammation than use of either agent alone. These data lend support to clinical observations in a murine model that showed the addition of systemic steroids to antimicrobials may aid in the treatment of severe MP infections. The specific and comparative effects of treatment with macrolides, systemic steroids, or their combination in patients with MP respiratory tract infections have not been fully investigated, and the effects of systemic steroids on infection-induced airway inflammation and airway function are not completely understood, particularly as they relate to infectious asthma. [14–16, 20, 21]

It remains to be investigated whether refractory and common MP pneumonia differ in their responses to macrolides. Therefore, the present study aims to investigate the cytokines related to each response mechanism and to identify the initial biomarkers that may distinguish common and refractory MP pneumonia at hospital admission.

Additionally, we investigated the critical cut-off values of the relevant biomarkers, including LDH, ferritin, IL-6, TNF-α, IL-17, and IL-18 to identify those that may be useful for the differential diagnosis of refractory and common MP pneumonia at hospital admission.

## Methods

### Subjects

The cohort included children with MP pneumonia admitted to the Department of Pediatrics at Hanyang University Guri Hospital in Seoul, Korea, between October 2016 and March 2017. At hospital admission, all children received physical examinations and routine laboratory tests; they were assessed for clinical symptoms such as fever (temperature > 38.5 °C), paroxysmal dry hacking cough with or without sputum, and abdominal pains or skin eruptions; chest radiographs were analyzed.

**Fig. 1:**
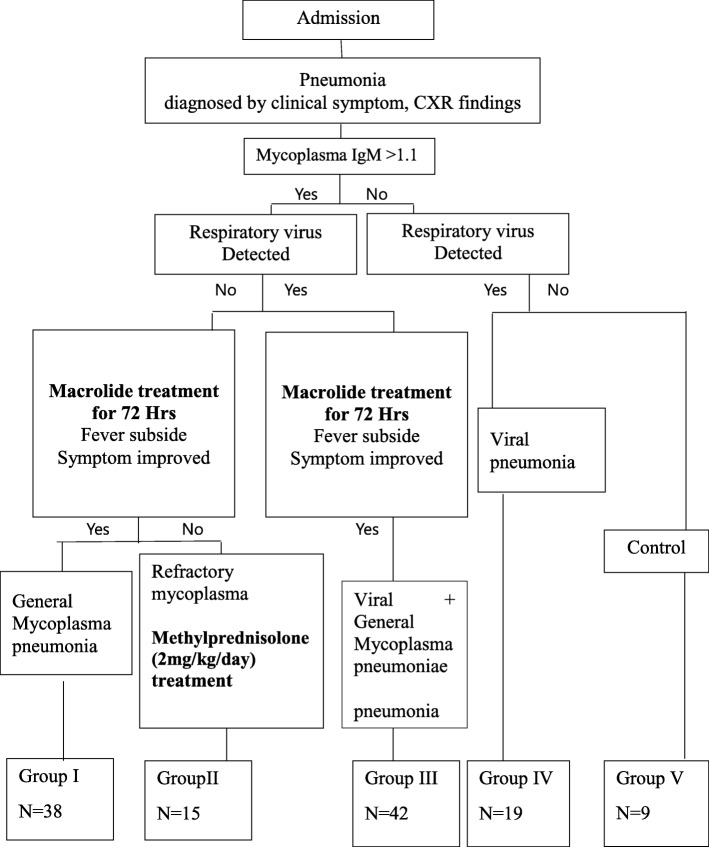
Classification of children into 5 groups

Written informed consent was obtained from all parents allowing the patients to undergo serum blood tests, respiratory virus examinations, and mycoplasma IgM tests, and for serum samples to be saved for later evaluation of biomarkers and cytokines. Patients were classified according to pneumonia type. For patients with MP pneumonia, treatment was initiated with macrolide antibiotics such as clarithromycin. After 72 h the patients were checked for reduction of fever, cough, and crackles on the lung, as well as improvement of radiological findings. If there was no improvement, the patient was considered to have refractory MP pneumonia. Then we start systemic steroid as additional treatment for prevent sequela of the lung in refractory MP pneumonia patients. Adequate dose of systemic steroid for refractory MP pneumonia is not established. In many other studies, different types of steroids were used in different doses. [14–16, 22] We used methylprednisolone as anti-inflammatory dose (2 mg/kg/day) (Fig. 1).

All children were discharged from the hospital with improved symptoms. After recovery and hospital discharge, the children were classified into five groups (Fig. 1, Table 1): those with MP pneumonia without any documented viral infection who had received macrolides alone (Group I), those with MP pneumonia without any documented viral infection who had received combination therapy with macrolides and methylprednisolone (Group II), those with MP pneumonia and documented viral infection who had received only macrolide antibiotics (Group III), and those with viral pneumonia without documented MP infection who had received empirical antibiotics and conservative treatment (Group IV). Controls were recruited from among children admitted due to pneumonia but who did not present any documented bacterial or viral infections (including MP) and had received antibiotics and conservative treatment (Group V). We further subdivided these five groups into Group A (including Groups I, III, IV, and V) and Group B (Group II), according to whether there was a response to macrolide treatment. The study protocol and consent forms were approved by the Institutional Review Board at Hanyang University (approval number 2016–03-009).

**Table 1:** Patient characteristics

	Group I	Group II	Group III	Group IV	Group V
N	38	15	42	19	9
Median age (yr)	7	5	4	5	5
Sex (M:F)	21:17	9:6	25:17	11:8	4:5

Group I: general MP pneumonia; Group II: refractory MP pneumonia; Group III: general MP pneumonia + viral pneumonia; Group IV: viral pneumonia; Group V: control

### Diagnosis of MP pneumonia

A diagnosis of MP pneumonia was based on clinical symptoms such as fever (> 38.5 °C), paroxysmal dry hacking cough with dyspnea, and crackles, as well as on the results of radiological findings and serological tests. Rather than using the classic particle agglutination test, an indirect enzyme-linked immunosorbent assay kit (Vircell, Granada, Spain) was used to identify MP as the causative organism of pneumonia. The antigen, composed of an extract of inactivated MP, was bound to the solid phase and incubated with diluted patient serum samples that blocked the IgG antibodies while the specific IgM proteins were bound to the antigen. After washing to eliminate unreacted proteins, samples were incubated with a conjugate consisting of anti-human IgM monoclonal antibodies and peroxidase. Conjugates that did not react were eliminated, and a peroxidase substrate was added. The blue color that developed was proportional to the concentration of specific antibodies present in the test serum. The results were expressed as an index (ratio between the optical density of the test sample and that of the cut-off value) that could be used as a quantitative measure, as it was proportional to the amount of specific IgM present. The test serum was considered positive when the ratio was > 1.1.

Previous studies have shown that there is no significant difference between the sensitivity and specificity of the enzyme-linked immunosorbent assay and those of the particle agglutination test. [23]

### Diagnosis of viral pneumonia

A diagnosis of viral pneumonia was based on respiratory symptoms, positive radiographic findings, and a polymerase chain reaction analysis of nasopharyngeal secretions positive for respiratory viruses. At admission, samples of nasopharyngeal secretions were obtained from the nasopharynx via aspiration through catheters after injection of 5 mL of sterile physiological saline solution into mucus traps. Each specimen was independently screened for the presence of common respiratory viruses, including influenza viruses, parainfluenza viruses, human coronaviruses, human rhinoviruses, human metapneumoviruses, human adenoviruses, and respiratory syncytial viruses, with reverse transcription-polymerase chain reaction using a commercial kit (Seeplex RV 12 ACE Detection, Seegene, Seoul, Korea).

### Measurement of serum LDH, ferritin, IL-6, IL-17, IL-18, and TNF-α levels

Venous blood was obtained from each subject. The average time between blood sampling obtained and the onset of disease symptom was (3.73 ± 1.42 days). Obtained blood sample was centrifuged at 1000×*g* for 10 min at 4 °C, and stored at − 70 °C. Serum LDH, ferritin, IL-6, IL-17, IL-18, and TNF-α levels were measured using commercial enzyme-linked immunosorbent assays (DBD Inc., San Diego, CA, USA) according to the manufacturer’s instructions. The detection limit was 2 pg/mL for IL-6, IL-17, IL-18, and TNF-α.

### Statistical analysis

Statistical analyses were performed using SPSS version 19.0 (SPSS Inc., Chicago, IL, USA). The data are expressed as median and IQR (Inter Quantile range). Statistical comparisons were made using Kruskal Wallis test and Mann-Whitney U test, Significance Probability (*P* value) of < 0.05 was considered statistically significant. A Kruskal Wallis test was used to compare concentrations of serum IL-6, IL-17, IL-18, TNF-α, LDH, and ferritin among the each 5 study groups. And Mann-Whitney U test was used to compare concentrations of serum IL-6, IL-17, IL-18, TNF-α, LDH, and ferritin among the 2 study groups (refractory MP vs Other pneumonia). The critical cut-off values of refractory MP pneumonia initial biomarkers were compared through receiver operating characteristic (ROC) curve analysis.

## Results

### Patient characteristics

A total of 123 children with pneumonia admitted to the Department of Pediatrics at Hanyang University Guri Hospital were included. The median age of the children was 5 years (range, 1–17 years). Table 1 presents the demographic characteristics of the children within each of the five groups. No differences were found among groups regarding the sex or age of the children.

Polymerase chain reactions revealed the presence of several viral infections within Groups III and IV, including 26 cases of rhinovirus, 15 cases of influenza, 5 cases of parainfluenza, 10 cases of respiratory syncytial virus, 7 cases of adenovirus, and 5 cases of coronavirus in Group III, and 12 cases of rhinovirus, 8 cases of influenza, 3 cases of parainfluenza, 5 cases adenovirus, and 3 cases of respiratory syncytial virus in Group IV. Some patients presented double viral infections.

### Serum LDH concentration

**Fig. 2:**
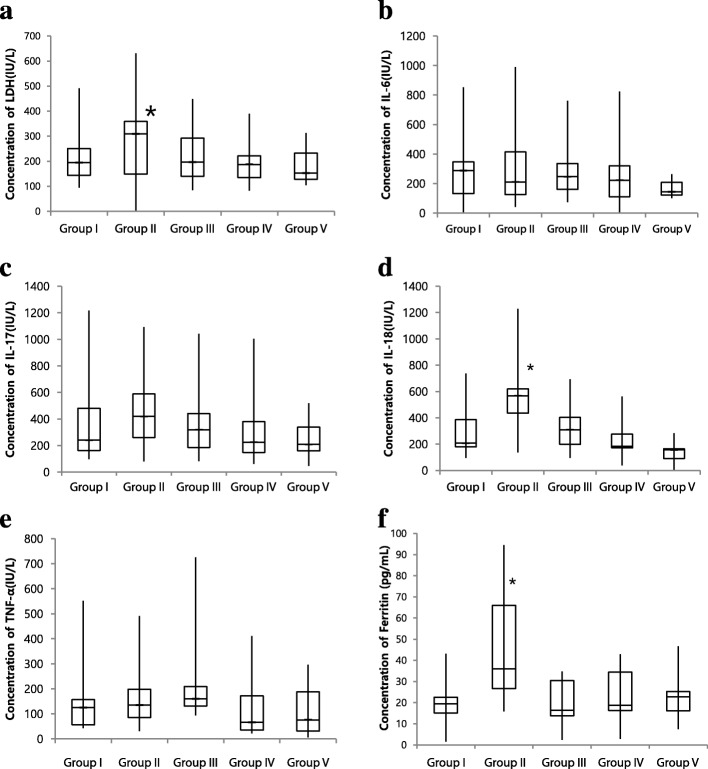
Serum concentrations of the biomarkers in groups I-V. There are increased the concentrations of LDH A(**a**), IL-18 (**b**), and ferritin (**f**) compared with them of the other groups. *P < 0.05 compared with the other groups. Group I: general MP pneumonia; Group II: refractory MP pneumonia; Group III: general MP pneumonia + viral pneumonia; Group IV: viral pneumonia; Group V: control

**Fig. 3:**
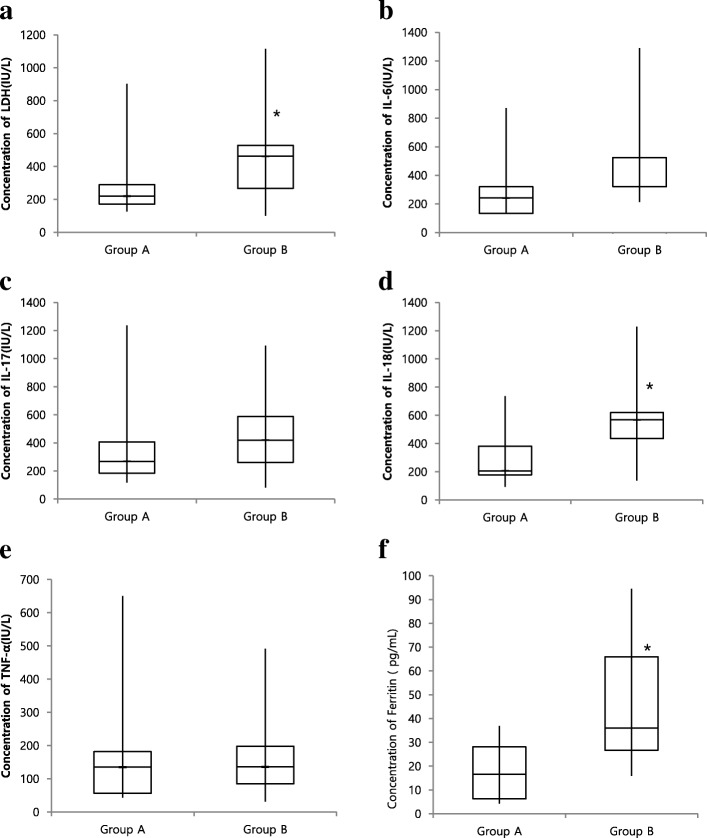
Serum concentrations of the biomarkers in groups A and B. Five groups subdivided into Group A (including Groups I, III, IV, and V) and Group B (Group II), according to whether there was a response to macrolide treatment. There are increased the concentration of LDH (**a**), IL-18 (**d**), ferritin (**f**) compared with Group A. * P < 0.05 compared with group A

The serum LDH concentration in Group II (median: 325.73 IU/L) was significantly higher than those in Groups I, III, IV, and V (median: 185.91, 201.17, 199.42 and 121.28 IU/L, respectively; all *P* < 0.05). (Fig. 2a) Furthermore, a comparison of serum LDH concentrations showed that the concentration in Group II was significantly higher when the five study groups were subdivided into Group A (Groups I, III, IV, V) and Group B (group II) (Fig. 3a).

### Serum IL-17 concentration

The serum IL-17 concentration in Group II (median: 426.08 pg/mL) was higher than in Groups I, III, IV, and V (226.06, 376.66, 203.39 and 219.48 pg/mL, respectively; all *P* < 0.05). However, there was no significant difference between the IL-17 level in Group II and those of the other groups (Fig. 2c). No significant difference was found between the IL-17 levels in Groups A and B. (Fig. 3c).

### Serum IL-18 concentration

The serum IL-18 concentration measured in Group II (median: 585.65 pg/mL) was significantly higher than in Groups I, III, IV, and V (median: 220.78, 297.15, 222.93 and 214.25 pg/mL, respectively; all *P <* 0.05; Fig. 2d). IL-18 concentration was significantly higher in Group B than in Group A (Fig. 3d).

### Serum ferritin concentration

The serum ferritin concentration measured in Group II (median: 35.95 pg/mL) was significantly higher than that in Groups I, III, IV, and V (median: 19.44, 14.15, 18.75, and 22.77 pg/mL, respectively; *P* < 0.05; Fig. 2f). The concentration of serum ferritin concentration in Group B was significantly higher than that of Group A (Fig. 3f).

### Serum IL-6 and TNF-α concentrations

No significant differences were found among groups regarding the concentrations of serum IL-6 and TNF-α (Fig. 2b,e)(Fig. 3b,e).

### Critical cut-off values

The cut-off values for the biomarkers and cytokines that were elevated in patients with refractory MP pneumonia (Group II) (i.e., LDH, IL-18, and ferritin), were calculated using ROC curve analysis**.** The analysis revealed that when the serum LDH concentration was 350 IU/L or higher, the sensitivity and specificity were 80 and 90%, respectively; when the serum IL-18 level was 430 pg/mL or higher, the sensitivity and specificity of a diagnosis of MP pneumonia were 73 and 80%, respectively; and when the serum ferritin concentration was 230 pg/mL or higher, the sensitivity and specificity were both 67% (Table 2).

**Table 2:** Critical cut-off values

Critical cut-off value	Sensitivity (95% CI)	Specificity (95% CI)
LDH (IU/L)		
310	73%	76%
330	73%	78%
350	73%	80%
370	67%	94%
390	60%	98%
IL-18 (pg/mL)		
340	93%	69%
360	93%	70%
380	80%	76%
400	80%	83%
430	80%	90%
Ferritin (pg/mL)		
190	67%	50%
200	67%	53%
210	67%	57%
220	67%	62%
230	67%	67%

*CI* confidence interval; *LDH* Lactate dehydrogenase; *IL*-*18* Interleukin 18

## Discussion

This study investigated the biomarkers and cytokines that distinguish common and refractory MP pneumonia at hospital admission. We found that the levels of serum LDH, IL-18, and ferritin were increased only in the refractory MP group, indicating that these are relevant biomarkers for diagnosing refractory MP pneumonia.

In this study, for patients with MP pneumonia, treatment was initiated with macrolide antibiotics such as clarithromycin. After 72 h, the patients were checked for reduction of fever, cough, and crackles on the lung, as well as improvement of radiological findings. If there was no improvement, the patient was considered to have refractory MP pneumonia. Although there is no exact definition of refractory MP pneumonia at present, guidelines and some studies define refractory MP pneumonia as no improvement in clinical and laboratory findings after 7 days of macrolides treatment. [22]

The detection of treatment failure of antibiotics is mostly based on objective clinical criteria, but also on the less well-defined subjective decisions of the treating physicians. Assessment of response should be performed early enough to allow successful rescue therapy, but not too early, to allow evidence of clinical response. The time to an antibiotic response depends on the site of infection, the pathogen and the patient’s immunological status, and different symptoms and signs may require different time points for evaluation, but usually 48 to 72 h were need for proper treatment. Therefore, there was defined 72 h as a standard of diagnosing refractory MP pneumonia in this study. [24] We considered treatment failure in the absence of symptomatic improvement 72 h after macrolide antibiotic treatment and added other treatments for prevent sequela of the lung. It was a systemic steroid.

MP infections occur annually worldwide; a recent MP epidemic occurred in 2015. The infections typically begin to spread during the autumn and remain for several months. The MP infection starts with the adherence of the bacteria to a host cell, which triggers the disease-related symptoms. The cytokines affected by the host immune response further contribute to the symptoms. The first-line antibiotic therapy for MP pneumonia is macrolide antibiotics. However, the number of pediatric patients with macrolide-refractory MP pneumonia increased noticeably in Korea when MP pneumonia was prevalent in 2015. Defining refractory MP is difficult, because few studies about the disease have been published. Although many studies are now underway, consensus about the treatment protocol for refractory MP has yet to be reached.

In a previous study, Tamura et al. confirmed that children with refractory MP pneumonia had cytopenia as well as increased LDH and ferritin levels.^2^ They also demonstrated that methylprednisolone pulse therapy improved radiological abnormalities as well as other symptoms, including fever. Hirao et al. (2011) reported that increased levels of IL-6 and TNF-α in acute MP pneumonia are associated with inflammation and described key roles of the many factors.^14^ Furthermore, Inamura et al. (2014) measured increased levels of LDH, aspartate aminotransferase, alanine aminotransferase, IL-18, and other factors in refractory MP infection.^12^ They suggested that these serum levels would be useful markers for the evaluation of therapeutic efficacy in refractory MP pneumonia and could be used as criteria to decide which patients are candidates for corticosteroid therapy.

Although these studies showed that parameters are significantly elevated in refractory MP infection and are dramatically decreased by corticosteroid therapy, additional studies focusing on the various inflammatory mediators are required to elucidate their mechanisms of action; this knowledge would aid in determining the appropriate initial therapy.

In the present study, we focused on the levels of LDH, ferritin, IL-6, IL-17, IL-18, and TNF-α, which are considered the primary factors in refractory MP pneumonia based on the studies presented above. We also subdivided the groups of patients with MP pneumonia into two groups, one comprised of patients who responded well to macrolide antibiotics and another comprised of patients who received corticosteroid therapy after a lack of response to macrolides. We then compared the levels of these factors between the subgroups. Our results showed significant differences in LDH, IL-18, and ferritin levels between pediatric patients with refractory and non-refractory MP pneumonia.

We also determined the cut-off value of each enzyme and cytokine to be used in the diagnosis of refractory MP. Determination of the cut-off values with appropriate sensitivity and specificity was required in order to apply our findings in a clinical setting. Enzyme and cytokine levels above the cut-off values can be used as indicators of refractory MP pneumonia, allowing for prompt initiation of adequate treatment.

Previous studies have investigated the enzymes and cytokines that are elevated in patients with refractory MP pneumonia. However, not all diagnostic biomarkers can provide information about the best course of treatment. Therefore, we investigated for the first time the biomarkers that predict the response to macrolide therapy. Although the investigated enzymes and cytokines can also be elevated in other severe infections, when measured together, they are useful for the diagnosis of refractory MP pneumonia.

The present study had some limitations. First, the number of patients was relatively low, which caused the group sizes to be small. Second, we did not account for the possibility of coinfections with other bacteria or viruses. Third, We could not perform sputum culturing and confirm macrolide - resistant *Mycoplasma pneumoniae*, because it was difficult to obtain a test sputum specimen in pediatric patients.

## Conclusions

We found that only patients with refractory MP pneumonia presented elevated levels of serum LDH, IL-18, and ferritin at hospital admission. This suggests that, taken together, these biomarkers may be a useful tool to distinguish between common and refractory MP pneumonia, as well as between MP and viral pneumonia at hospital admission. These findings should allow for other appropriate therapies for refractory MP pneumonia to be combined with macrolide treatment and administered earlier. In order to use these results clinically, it is necessary to continue study on a larger number of subjects.

## Data Availability

The datasets used and analyzed during the current study are available from the corresponding author on reasonable request.

## Abbreviations

IL-17: interleukin 17
IL-18: Interleukin 18
IL-6: interleukin 6
LDH: Lactate dehydrogenase
MP: *Mycoplasma pneumoniae*
ROC: receiver operating characteristic
Th: T-helper
TNF-α: tumor necrosis factor-α
